# Exploring the Potential and Limitations of Chat Generative Pre-trained Transformer (ChatGPT) in Generating Board-Style Dermatology Questions: A Qualitative Analysis

**DOI:** 10.7759/cureus.43717

**Published:** 2023-08-18

**Authors:** Ibraheim Ayub, Dathan Hamann, Carsten R Hamann, Matthew J Davis

**Affiliations:** 1 Dermatology, A.T. Still University School of Osteopathic Medicine, Mesa, USA; 2 Dermatology, Dermatology Residency, HonorHealth, Scottsdale, USA; 3 Dermatology, HonorHealth Dermatology Residency, Scottsdale, USA; 4 Dermatology, Dartmouth-Hitchcock Medical Center, Lebanon, USA

**Keywords:** multiple-choice questions, artificial intelligence in medicine, medical education, chatgpt, dermatology

## Abstract

This article investigates the limitations of Chat Generative Pre-trained Transformer (ChatGPT), a language model developed by OpenAI, as a study tool in dermatology. The study utilized ChatPDF, an application that integrates PDF files with ChatGPT, to generate American Board of Dermatology Applied Exam (ABD-AE)-style questions from continuing medical education articles from the *Journal of the American Board of Dermatology*. A qualitative analysis of the questions was conducted by two board-certified dermatologists, assessing accuracy, complexity, and clarity. Out of 40 questions generated, only 16 (40%) were deemed accurate and appropriate for ABD-AE study preparation. The remaining questions exhibited limitations, including low complexity, lack of clarity, and inaccuracies. The findings highlight the challenges faced by ChatGPT in understanding the domain-specific knowledge required in dermatology. Moreover, the model's inability to comprehend the context and generate high-quality distractor options, as well as the absence of image generation capabilities, further hinders its usefulness. The study emphasizes that while ChatGPT may aid in generating simple questions, it cannot replace the expertise of dermatologists and medical educators in developing high-quality, board-style questions that effectively evaluate candidates' knowledge and reasoning abilities.

## Introduction

Chat Generative Pre-trained Transformer (ChatGPT) is a language model developed by OpenAI (San Francisco, CA, USA) that has shown promise in various natural language processing (NLP) tasks, including medical education and multiple-choice question generation [1,2]. Within dermatology, ChatGPT has been shown to create case reports indistinguishable from those written by humans and assist in creating patient handouts [3,4]. Beyond these applications, the model holds promise in streamlining routine administrative duties, facilitating patient education, enhancing medical instruction, and promoting improved healthcare literacy among patients [5]. Furthermore, ChatGPT has been employed for taking licensing examinations and responding to specialty board review questions, demonstrating an average accuracy rate close to passing thresholds [6,7]. While promising, the use of ChatGPT in this context poses certain limitations and challenges. These include the potential to generate erroneous data or incorrect answers, as well as the risk of introducing biased content [5]. In this study, we explore the limitations of ChatGPT as a study tool in dermatology through a qualitative analysis of the ChatGPT-generated American Board of Dermatology Applied Exam (ABD-AE)- style questions.

## Materials and methods

**Figure 1:**
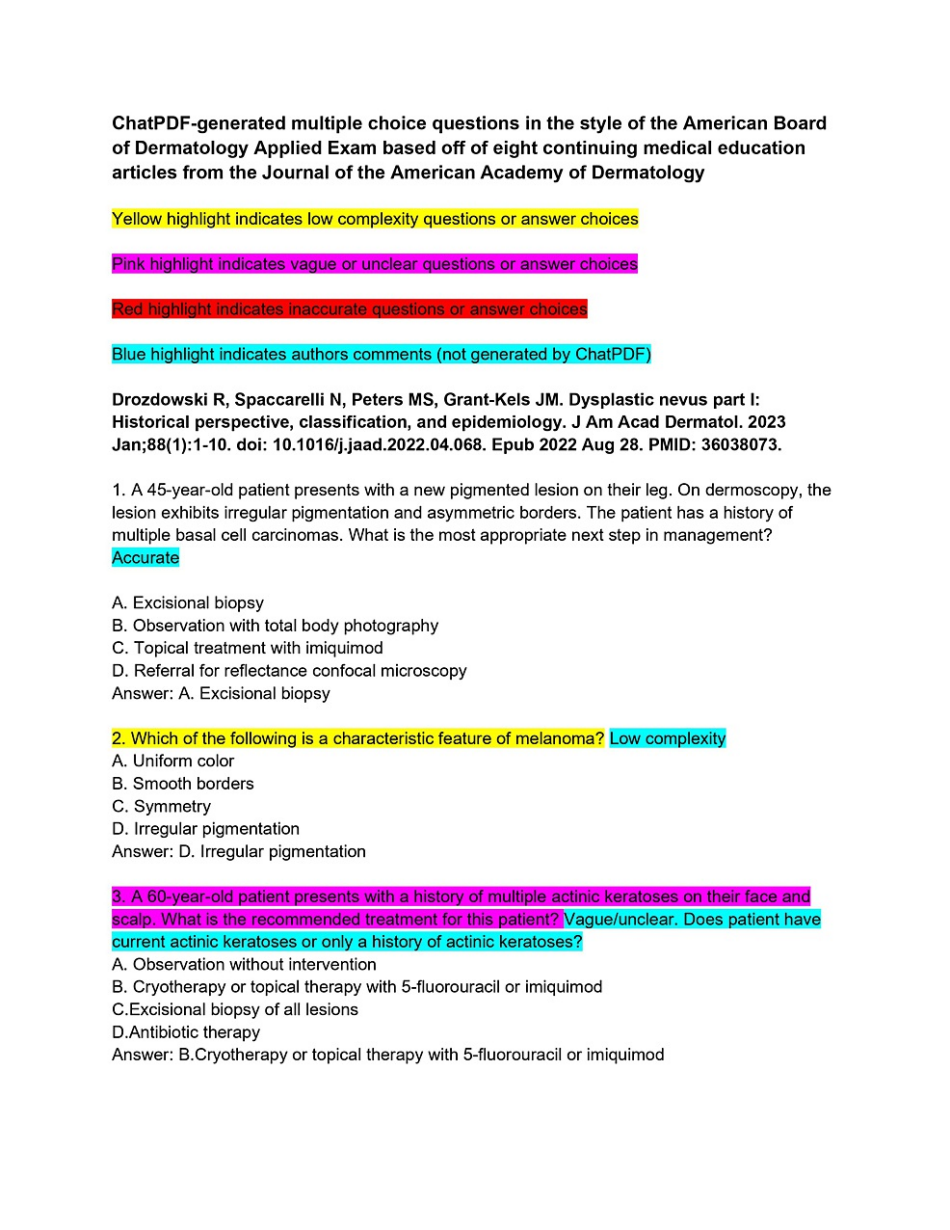
ChatPDF-generated multiple-choice questions in the style of the American Board of Dermatology Applied Exam based on eight continuing medical education articles from the Journal of the American Academy of Dermatology with author commentary (Questions 1-3).

**Figure 2:**
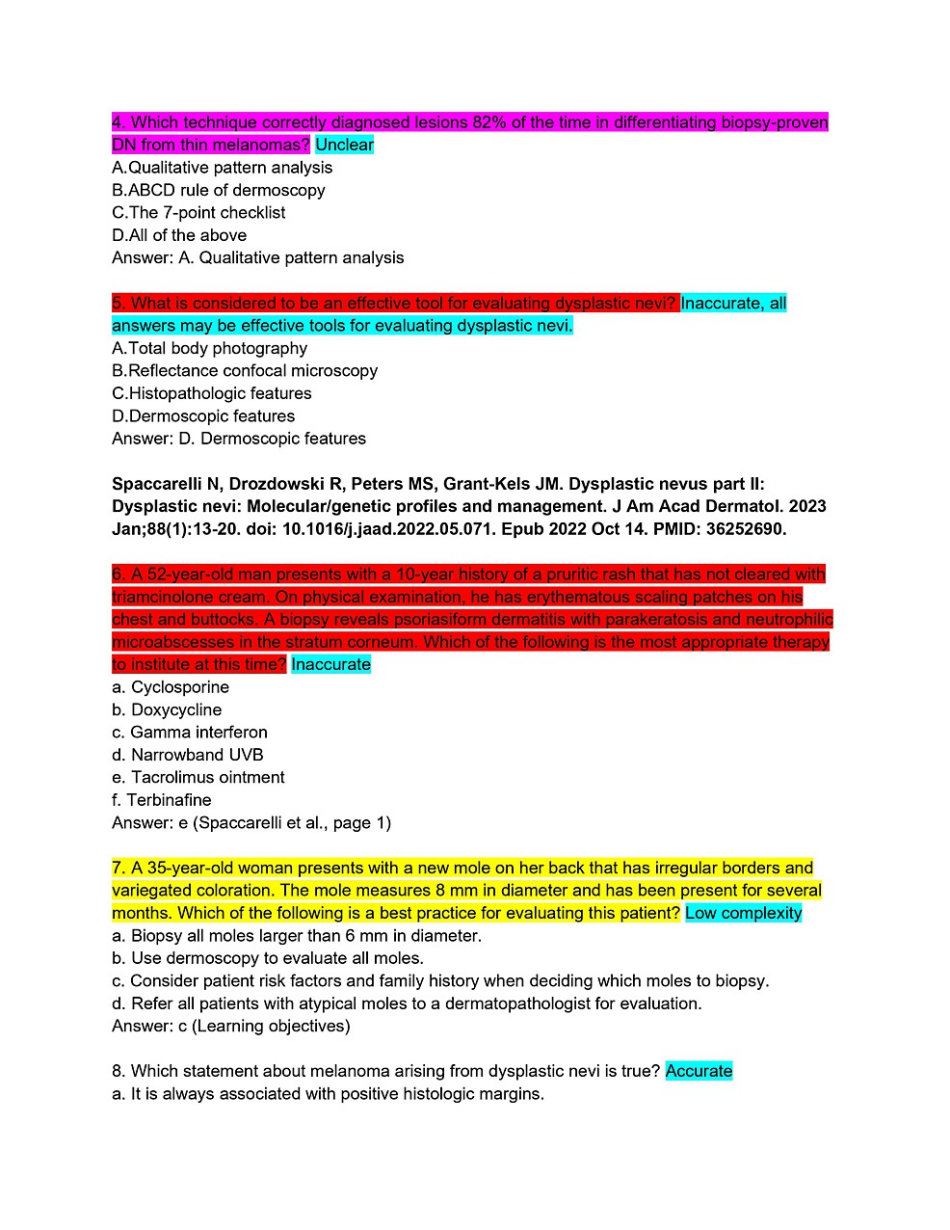
ChatPDF-generated multiple-choice questions in the style of the American Board of Dermatology Applied Exam based on eight continuing medical education articles from the Journal of the American Academy of Dermatology with author commentary (Questions 4-8).

**Figure 3:**
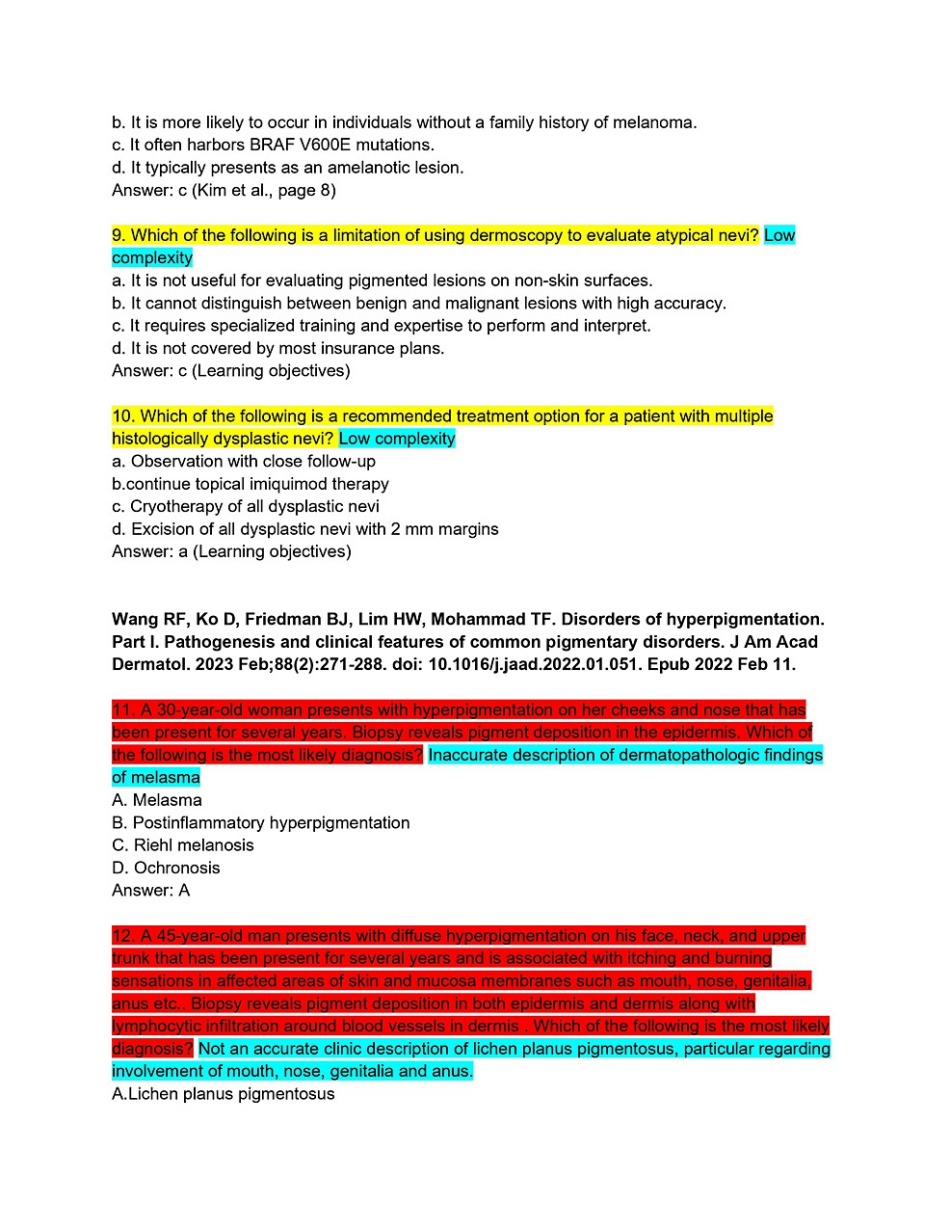
ChatPDF-generated multiple-choice questions in the style of the American Board of Dermatology Applied Exam based on eight continuing medical education articles from the Journal of the American Academy of Dermatology with author commentary (Questions 9-12).

**Figure 4:**
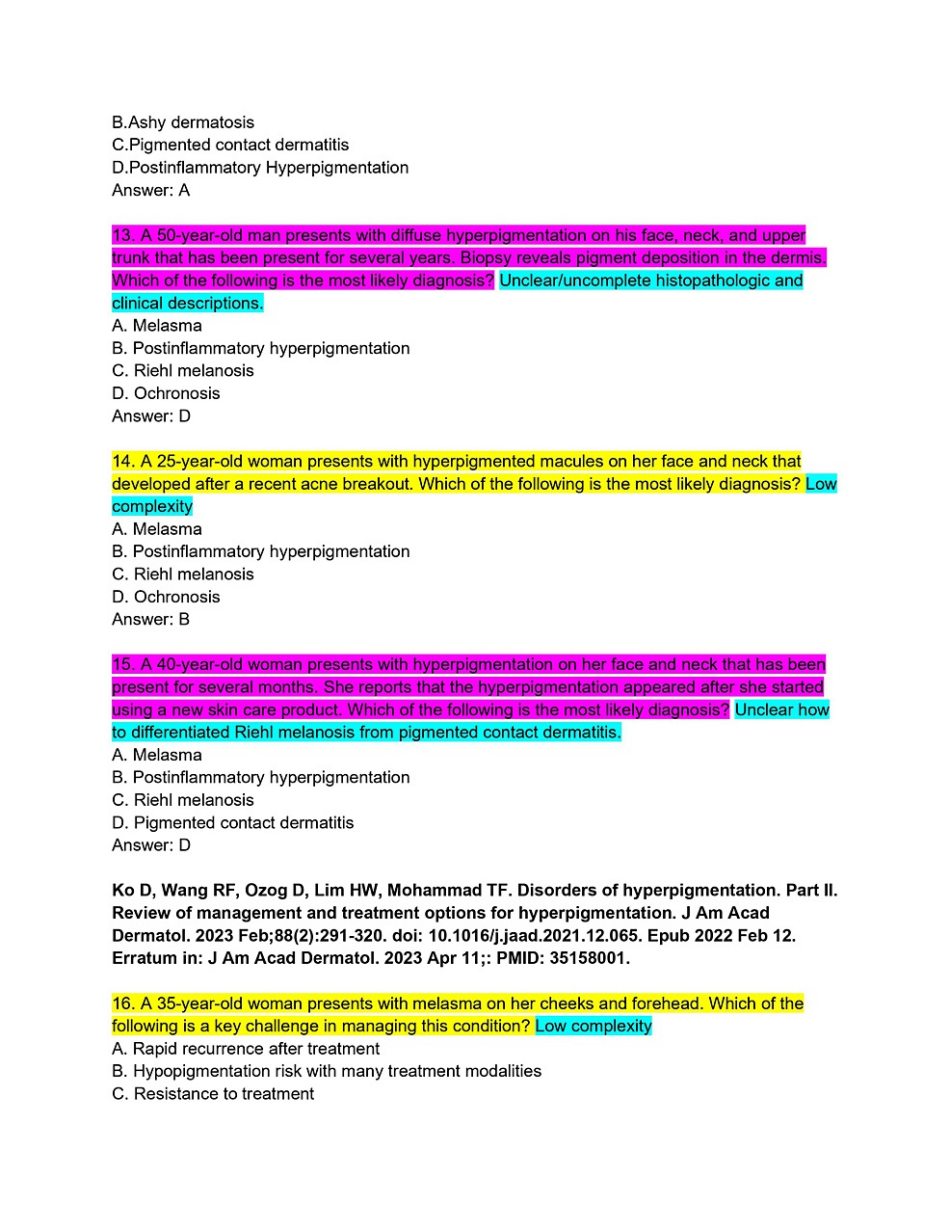
ChatPDF-generated multiple-choice questions in the style of the American Board of Dermatology Applied Exam based on eight continuing medical education articles from the Journal of the American Academy of Dermatology with author commentary (Questions 13-16).

**Figure 5:**
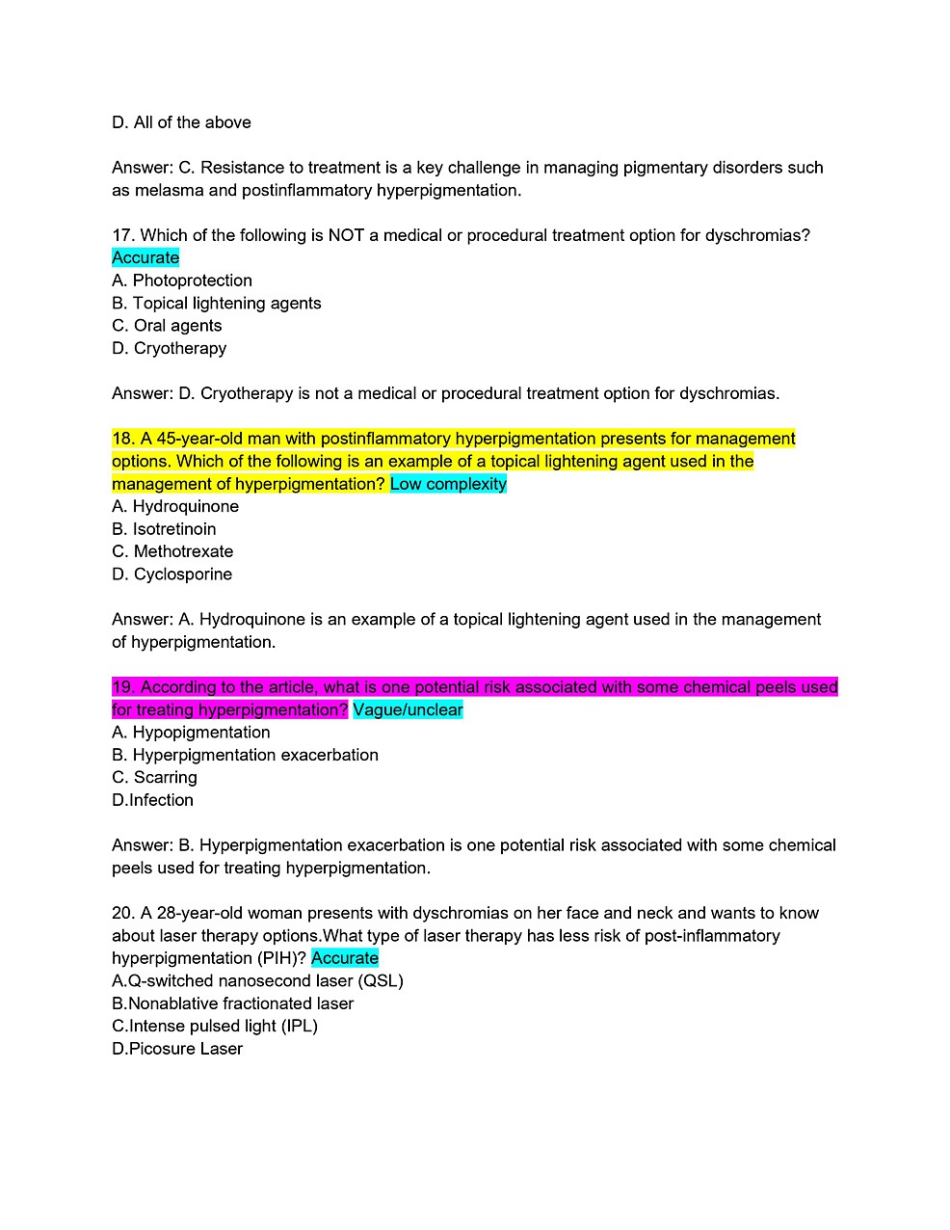
ChatPDF-generated multiple-choice questions in the style of the American Board of Dermatology Applied Exam based on eight continuing medical education articles from the Journal of the American Academy of Dermatology with author commentary (Questions 17-20).

**Figure 6:**
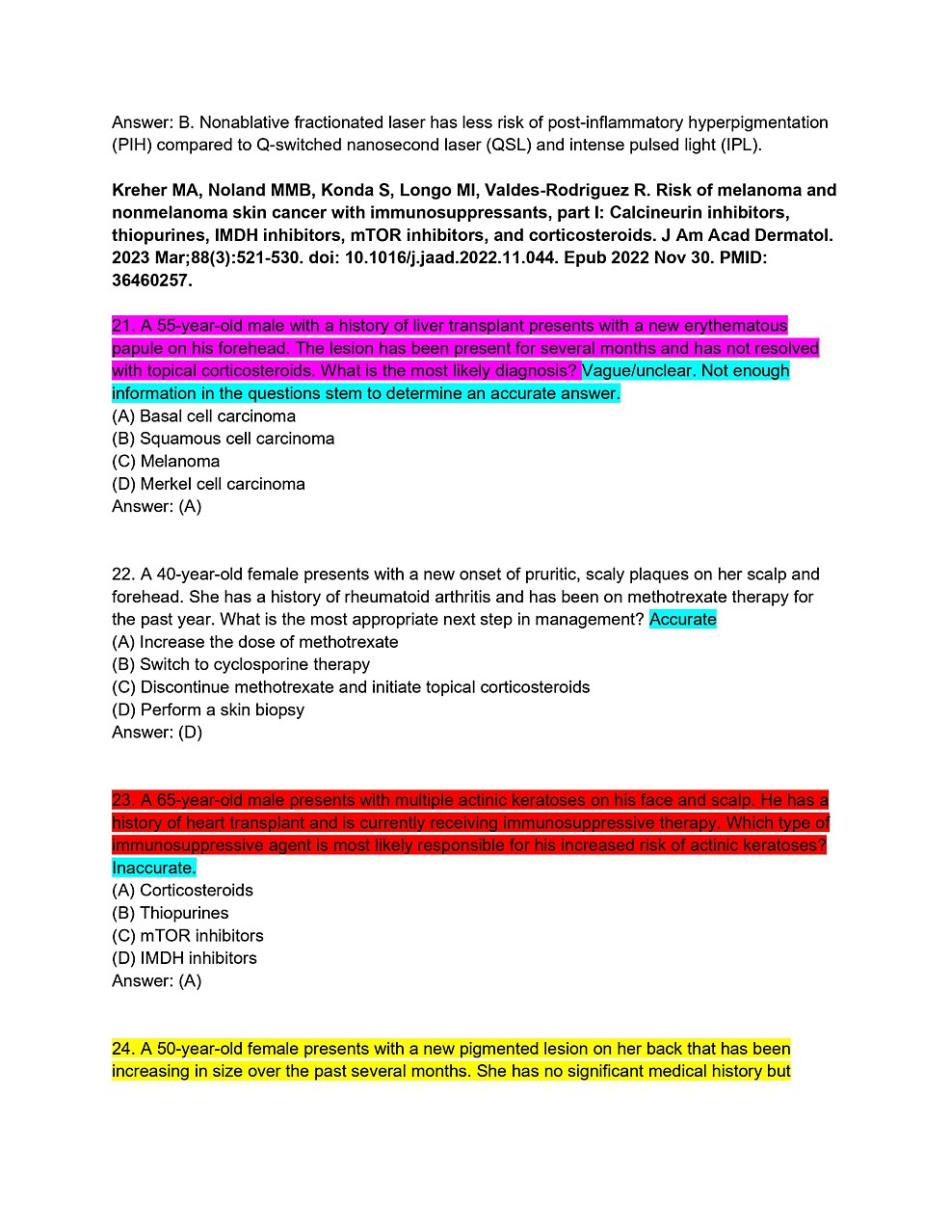
ChatPDF-generated multiple-choice questions in the style of the American Board of Dermatology Applied Exam based on eight continuing medical education articles from the Journal of the American Academy of Dermatology with author commentary (Questions 21-24).

**Figure 7:**
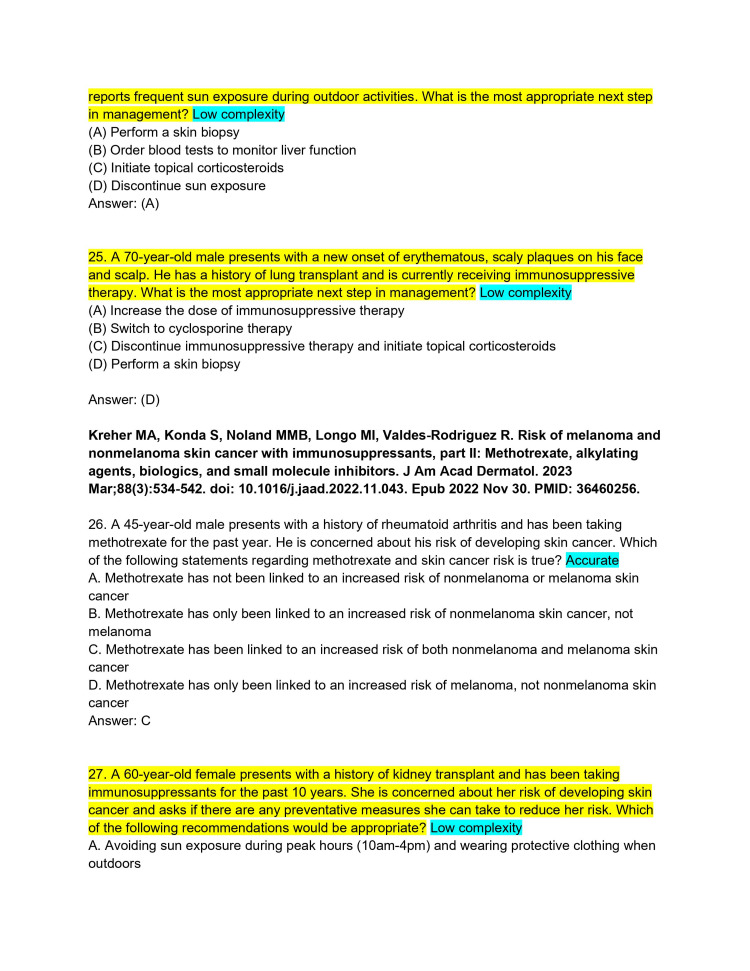
ChatPDF-generated multiple-choice questions in the style of the American Board of Dermatology Applied Exam based on eight continuing medical education articles from the Journal of the American Academy of Dermatology with author commentary (Questions 25-27).

**Figure 8:**
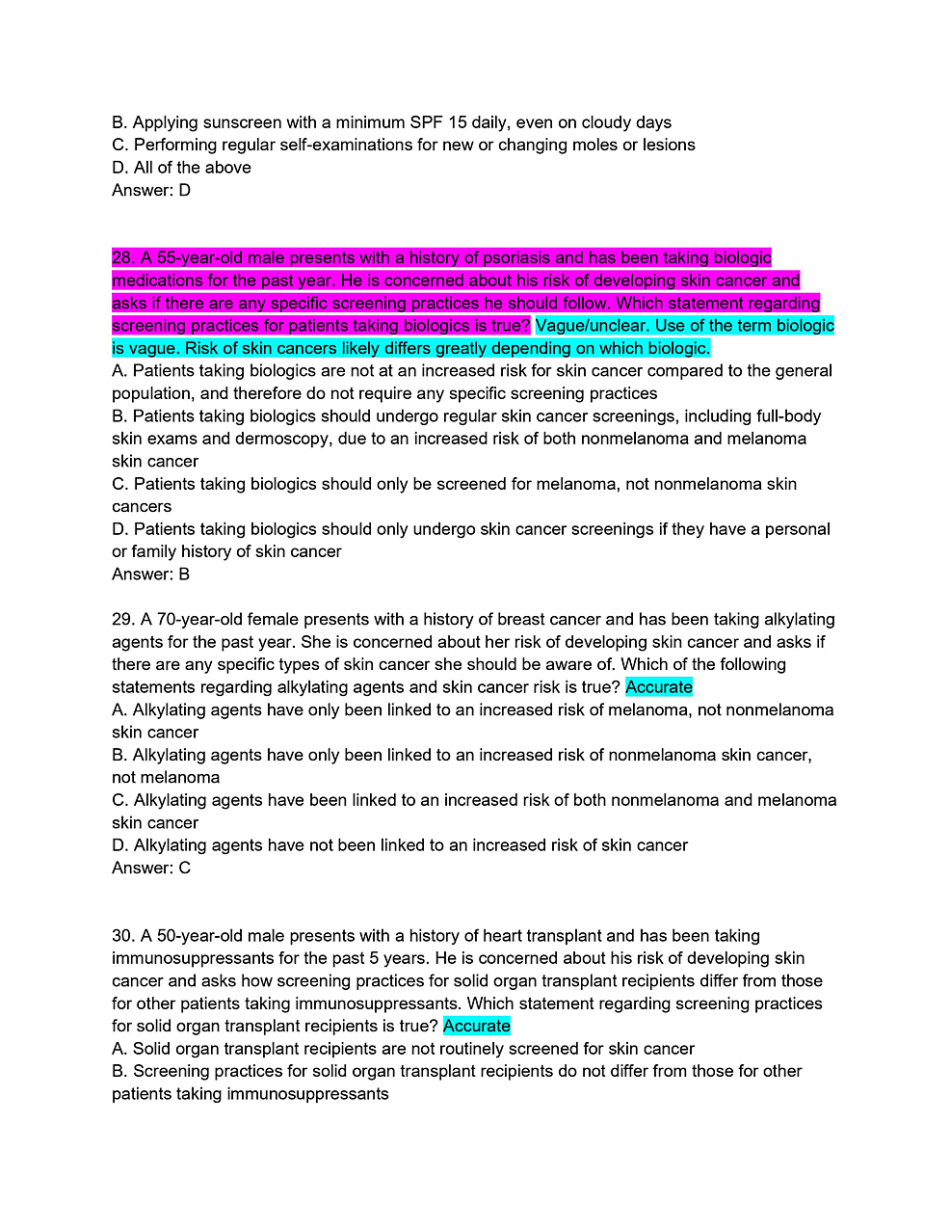
ChatPDF-generated multiple-choice questions in the style of the American Board of Dermatology Applied Exam based on eight continuing medical education articles from the Journal of the American Academy of Dermatology with author commentary (Questions 28-30).

**Figure 9:**
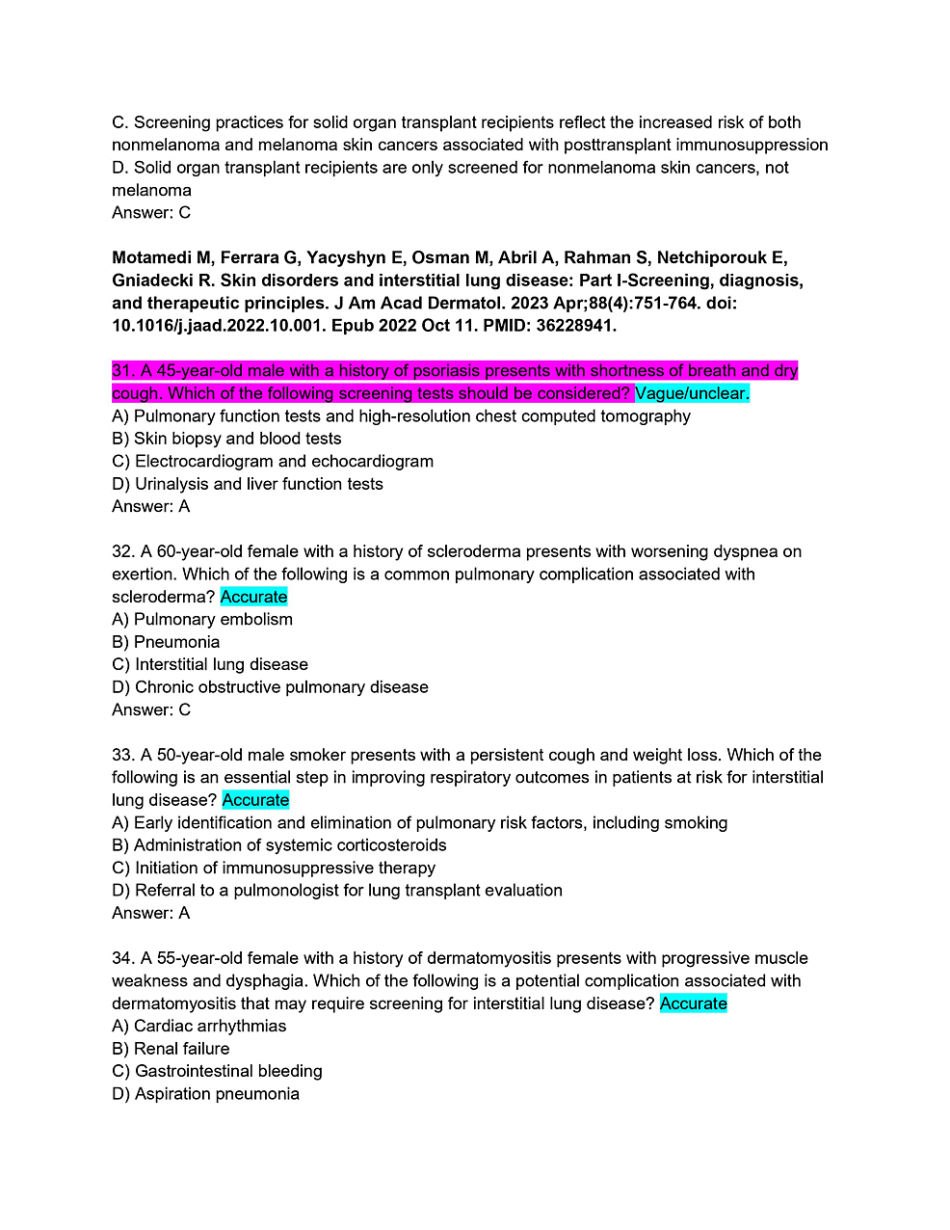
ChatPDF-generated multiple-choice questions in the style of the American Board of Dermatology Applied Exam based on eight continuing medical education articles from the Journal of the American Academy of Dermatology with author commentary (Questions 31-34).

**Figure 10:**
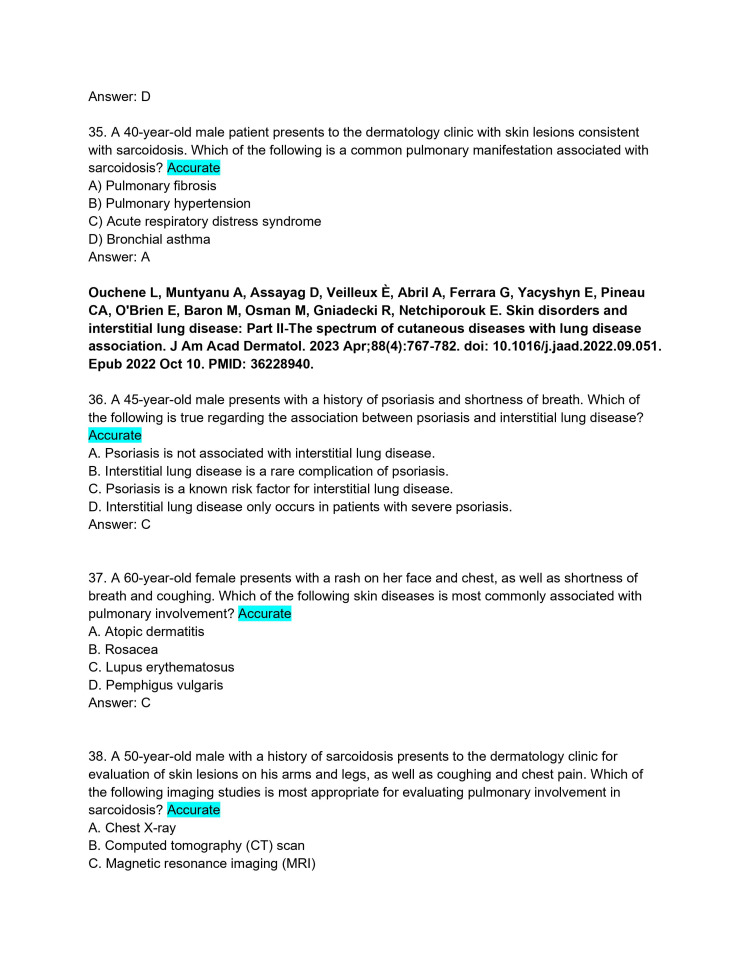
ChatPDF-generated multiple-choice questions in the style of the American Board of Dermatology Applied Exam based on eight continuing medical education articles from the Journal of the American Academy of Dermatology with author commentary (Questions 35-38).

**Figure 11:**
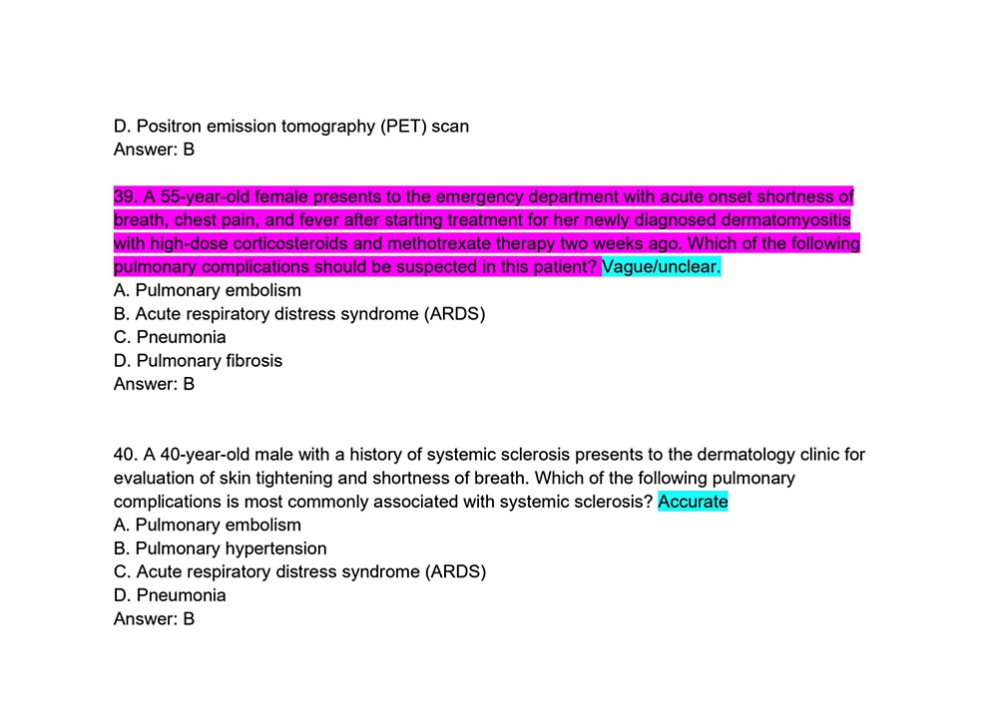
ChatPDF-generated multiple-choice questions in the style of the American Board of Dermatology Applied Exam based on eight continuing medical education articles from the Journal of the American Academy of Dermatology with author commentary (Questions 39-40).

ChatPDF is an application that combines the ability to upload entire PDF files into a ChatGPT 3.5 portal. The continuing medical education (CME) articles from the *Journal of the American Academy of Dermatology* (JAAD) are considered high-yield review material for the ABD-AE. CME articles from the JAAD (volume 88, issues 1-4) were imported into ChatPDF [8-15]. It was then asked to create five ABD-AE-style multiple-choice questions. The resulting sets of questions from each article were subjected to an independent and rigorous analysis by two board-certified dermatologists, ensuring a comprehensive evaluation of the questions' quality (Figures 1-11). The evaluation encompassed three essential dimensions: accuracy, complexity, and clarity. Dermatologists individually assessed each question's appropriateness for the required depth of knowledge for the ABD-AE, and the clarity of its wording and structure. The evaluation process involved in-depth discussions between the dermatologists to resolve any scoring discrepancies and to foster a consensus-driven evaluation.

## Results

**Figure 12:**
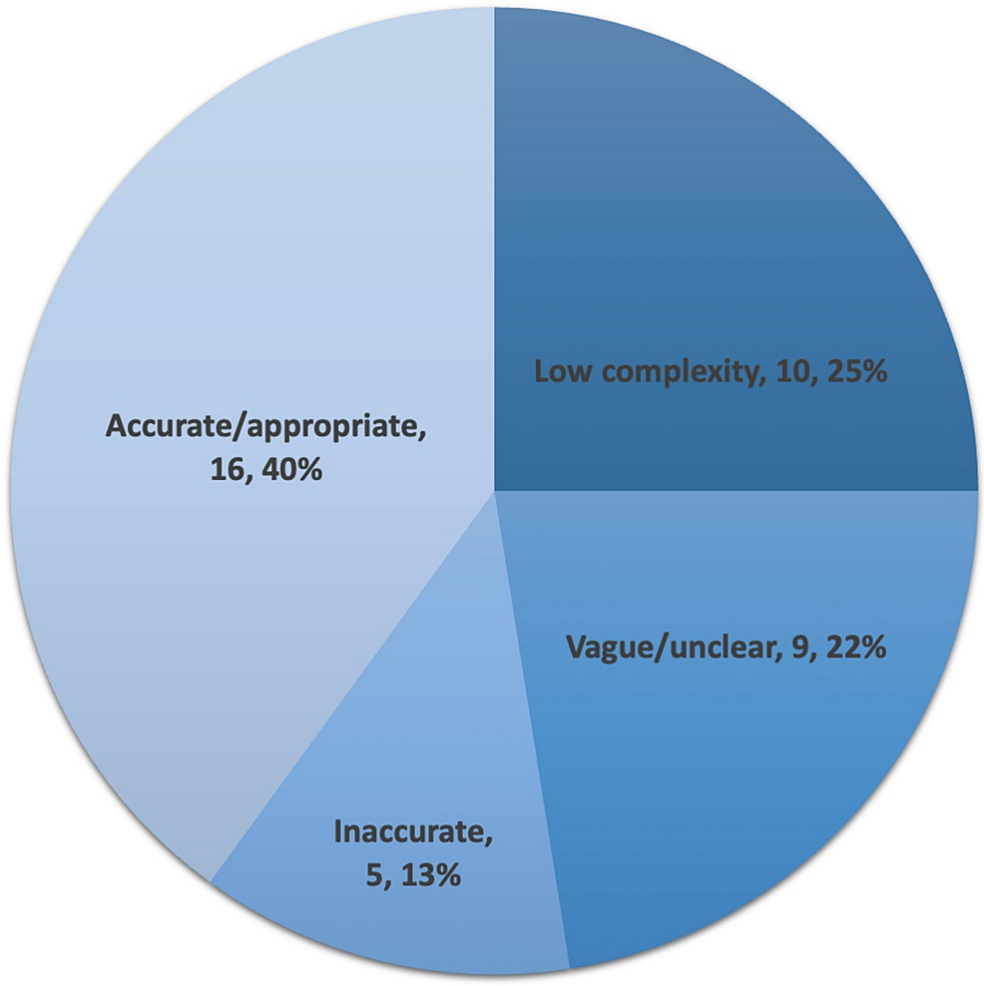
Pie chart depicting the categorization of ChatPDF-generated multiple-choice questions in the style of the American Board of Dermatology Applied Exam based on eight continuing medical education articles from the Journal of the American Academy of Dermatology.

A total of 40 questions were created using ChatPDF for the eight CME articles. After an independent review of the questions, it was found that out of 40 questions, 10 (25%) were of low complexity, 9 (22.5%) were vague or unclear, and 5 (12.5%) were inaccurate (Figure 12). Of the 40 questions, only 16 (40%) questions created using ChatGPT 3.5 were accurate and at an appropriate level of complexity for a trainee studying for ABD-AE (Table 1).

**Table 1:** Categorization of ChatPDF-generated multiple-choice questions in the style of the American Board of Dermatology Applied Exam based on eight continuing medical education articles from the Journal of the American Academy of Dermatology.

Article title	Low complexity	Vague/unclear	Inaccurate	Accurate/appropriate
Dysplastic nevus part I: historical perspective, classification, and epidemiology	1	2	1	1
Dysplastic nevus part II: dysplastic nevi: molecular/genetic profiles and management	3	0	1	1
Disorders of hyperpigmentation. Part I. Pathogenesis and clinical features of common pigmentary disorders	1	2	2	0
Disorders of hyperpigmentation. Part II. Review of management and treatment options for hyperpigmentation	2	1	0	2
Risk of melanoma and nonmelanoma skin cancer with immunosuppressants, part I: calcineurin inhibitors, thiopurines, IMDH inhibitors, mTOR inhibitors, and corticosteroids	2	1	1	1
Risk of melanoma and nonmelanoma skin cancer with immunosuppressants, part II: methotrexate, alkylating agents, biologics, and small molecule inhibitors	1	1	0	3
Skin disorders and interstitial lung disease: part I - screening, diagnosis, and therapeutic principles	0	1	0	4
Skin disorders and interstitial lung disease: part II -the spectrum of cutaneous diseases with lung disease association	0	1	0	4
Total, *n* (%)	10 (25)	9 (22.5)	5 (12.5)	16 (40)

## Discussion

ChatGPT has limitations as an educational tool for ABD-AE study preparation, with <50% of the generated questions found to be accurate and appropriate. The questions exhibited low complexity, as exemplified by inquiries like, "Which of the following is a characteristic feature of melanoma? A. Uniform color B. Smooth borders C. Symmetry D. Irregular pigmentation; Answer: D." Moreover, there were issues with clarity, such as the question, "A 45-year-old male with a history of psoriasis presents with shortness of breath and dry cough. Which of the following screening tests should be considered? A) Pulmonary function tests and high-resolution chest computed tomography B) Skin biopsy and blood tests C) Electrocardiogram and echocardiogram D) Urinalysis and liver function tests; Answer: A." Furthermore, 12.5% of generated questions were incorrect or inaccurate, raising concerns about the reliability of artificial intelligence-generated questions. This study identified the limited domain-specific knowledge of ChatGPT as a major limitation as dermatology requires a deep understanding of skin anatomy, physiology, and pathology, which ChatGPT lacks. ChatGPT's inability to understand the context and generate high-quality distractor options, as well as its incapacity to generate images, further limits its usefulness. To address these limitations, future research should focus on developing domain-specific language models that possess deep knowledge of dermatology. By improving the model's understanding of skin-related concepts and its ability to generate contextually appropriate questions and distractors, it may become a more reliable and valuable tool for medical education and exam preparation in dermatology.

## Conclusions

Our study demonstrates that while ChatGPT shows promise as an educational tool in dermatology, its limitations must be acknowledged. Generating ABD-AE-style questions with sufficient accuracy, complexity, and clarity remains a challenge for ChatGPT. The model's inability to understand context and lack of domain-specific knowledge contribute to the generation of suboptimal questions. Future research efforts addressing these shortcomings might increase its utility in question generation for the ABD-AE. In conclusion, while ChatGPT may help generate simple questions, it cannot replace the expertise of dermatologists and medical educators in developing high-quality, board-style questions that accurately test a candidate's knowledge and reasoning abilities.
